# Virological failure and antiretroviral resistance among HIV-infected children after five years follow-up in the ANRS 12225-PEDIACAM cohort in Cameroon

**DOI:** 10.1371/journal.pone.0248642

**Published:** 2021-03-18

**Authors:** Paul Alain Tagnouokam-Ngoupo, Ida Calixte Penda, Jules Brice Tchatchueng Mbougua, Suzie Tetang Ndiang, Francis Yuya Septoh, Angeladine Kenne, Jeannine Eboumbou Ngallè, Sorel Jakpou, Francis Ateba Ndongo, Josiane Warszawski, Albert Faye, Mathurin Cyrille Tejiokem, The ANRS-Pediacam Study Group

**Affiliations:** 1 Service de Virologie, Centre Pasteur du Cameroun, Membre du Réseau International des Instituts Pasteur, Yaoundé, Cameroun; 2 Department of Clinical Sciences, Faculty of Medicine and Pharmaceutical Sciences, University of Douala, Douala, Cameroon; 3 Hôpital Laquintinie de Douala, Douala, Cameroun; 4 Service d’Epidémiologie et de Santé Publique, Centre Pasteur du Cameroun, Membre du Réseau International des Instituts Pasteur, Yaoundé, Cameroun; 5 Service de Pédiatrie, Centre Hospitalier d’Essos, Yaoundé, Cameroun; 6 Unité Pédiatrique de Jour, Centre Mère et Enfant de la Fondation Chantal Biya, Yaoundé, Cameroun; 7 Center for Research in Epidemiology and Population Health U1018, Clinical Epidemiology, INSERM, Le Kremlin-Bicetre, France; 8 Université Paris-Sud, Public Health, Le Kremlin-Bicêtre, France; 9 Assistance Publique-Hôpitaux de Paris, Hôpital Bicêtre, Le Kremlin-Bicêtre, France; 10 Assistance Publique des Hôpitaux de Paris, Pédiatrie Générale, Hôpital Robert Debré, Paris, France; 11 Université Paris 7—Denis Diderot, Paris, Île-de-France, France; 12 INSERM UMR-S 1123 (ECEVE), Paris, France; University of Cincinnati College of Medicine, UNITED STATES

## Abstract

**Objective:**

In the present study, we aimed to evaluate the virological failure (VF) and drug resistance among treated HIV-infected children after five years follow-up in the ANRS-Pediacam cohort in Cameroon.

**Methods:**

From November 2007 to October 2011, HIV-infected children born to HIV-infected mothers were included in the ANRS-PEDIACAM study and followed-up for more than 5 years. Plasma viral load (VL) was measured at each visit (every three months until month 24 and every 6 months thereafter). VF was the main outcome and HIV drug resistance test was performed using the ANRS procedures and algorithm.

**Results:**

Data from 155 children were analyzed. The median age at combination antiretroviral therapy (cART) initiation was 4.2 months (interquartile range (IQR): 3.2–5.8), with 103 (66.5%) children taking LPV/r-containing regimen and 51 (32.9%) children taking NVP. After five years follow-up, 63 (40.6%; CI: 32.9–48.8) children experienced VF. The median duration between cART initiation and VF was 22.1 months (IQR: 11.9–37.1) with a median VL of 4.8 log10 (IQR: 4.0–5.5). Among the 57 children with HIV drug resistance results, 40 (70.2%) had at least one drug resistance mutation. The highest resistance rates (30.4–66.1%) were obtained with Lamivudine; Efavirenz; Nevirapine and Rilpivirine.

**Conclusions:**

These results show high resistance to NNRTI and emphasize the need of VL and resistance tests for optimal follow-up of HIV-infected people especially children.

## Introduction

In 2018, UNAIDS estimated that 37.9 million people were living with HIV worldwide. Among them, 1.7 million were children under 15 years with 160,000 newly infected mainly by vertical transmission [1]. An estimated 1.6 million new HIV infections among children have been averted, since 1995, with the use of antiretroviral (ARV) medicines in women living with HIV during pregnancy and breastfeeding [2]. About 54% of children living with HIV were receiving combination antiretroviral therapy (cART) in 2018 globally and more efforts are needed to scale up treatment in this vulnerable population [1, 3]. It is well known that early cART in children helps in improving immune reconstitution, decreasing AIDS-related mortality and millions of lives have been saved since the adoption of this treatment strategy [4, 5]. In Cameroon, many efforts have also been developed in order to reduce the HIV burden in children through the prevention of mother-to-child transmission (PMTCT) and one main strategy was the adoption and implementation of the option B+ in 2012 [6].

Despite the significant progress in improving access to ART in paediatric population in resource-limited settings (RLS), limited access to adapted drug formulations and stock out remain challenges for cART treatment success. Therefore, children in routine clinical care can experience sustained detectable viral replication even under potent combination therapy. Various factors account to this failure to achieve viral clearance, but drug resistance is the main factor with an impact observed not only at individual level but also at population level. Increased provision of antiretroviral therapy in sub-Saharan Africa has led to a growing number of children with treatment failure and acquired drug-resistant HIV ranging from 19.2% to more than 80% in some studies [7, 8]. Moreover, a high proportion (10–51%) of pre-treatment drug resistance is reported in naïve HIV-infected children in low- and middle-income countries including Cameroon [9–15].

The present work aimed to evaluate the virological failure and drug resistance in the paediatric population from the ANRS-Pediacam cohort study in Cameroon.

## Materials and methods

### Ethics

The ANRS–Pediacam study was approved by the National Ethics committee (N°038/CNE/DNM/07, 4th June 2007 and N°139/CNE/SE/2010, 20th August 2010) and administrative authorization was obtained from the Ministry of Public Health (D48-13/AAR/MINSANTE/SG/DROS/CRC/CEA1, 7th September 2007 and D30-438/ AAR/MINSANTE/SG/DROS/CRC/JA, 14th May 2012). Prior to inclusion, written informed consent was obtained from the child’s parent or guardian after information on the study objectives and procedures.

### Study population

Analysis was conducted based on data collected in the ANRS-PEDIACAM study as described elsewhere [16, 17]. Briefly, ANRS-PEDIACAM is an ongoing prospective observational study based in three referral hospitals in Cameroon: the Mother and Child Center of the Chantal Biya Foundation (MCC-CBF), and the Essos Hospital Center (EHC) in Yaounde, and the Laquintinie Hospital in Douala (LH) under the coordination of the Centre Pasteur of Cameroon (CPC). The study included HIV-infected children born to HIV-infected mothers from November 2007 to October 2011.

A total of 210 HIV-infected infants were included and cART was systematically offered when the HIV status was confirmed using PCR (Generic HIV DNA Cell, Biocentric, Bandol, France). The initial cART regimen followed the Cameroon National Guidelines recommending zidovudine (or stavudine for infants with anemia) and lamivudine associated with lopinavir/ritonavir if nevirapine (NVP) has been used for PMTCT, or otherwise with NVP [17]. During follow-up, switching of cART regimen was mainly due to modification of national recommendations, stock out or treatment failure. At each visit, a standardised questionnaire was used to collect data on demographic, clinical, anthropometrical, and therapeutic parameters by the physician, assisted by other health personnel (nurse, psychologist, or pharmacist). For each child, whole blood was drawn by venepuncture on EDTA tubes and routed to the Centre Pasteur of Cameroon for biological analyses.

### Viral load and drug resistance testing

Plasma viral load (VL) was measured at each visit (every three months until month 24 and every 6 months thereafter) using the m2000rt Real Time HIV-1 assay (Abbott Molecular, Des Plaines, IL, USA) or the Generic HIV Charge Virale (Biocentric, Bandol, France) [18]. The lower limits of detection were 40 copies/mL and 60 copies/mL for Abbott and Biocentric assays, respectively.

HIV drug resistance test was performed using the ANRS procedures and algorithm [19]. Briefly, RNA was extracted from 1 mL plasma samples using QIAmp viral RNA mini kit (QIAGEN, Courtaboeuf, France) followed by the amplification of Protease (Prot) (1–100 amino acids) and Reverse transcriptase (RT) (1–250 amino acids) regions of *pol* gene by nested RT-PCR as previously described [18, 20]. The amplified products were further sequenced using the Sanger’s method with CEQ Dye Terminator Cycle Sequencing and Quick Start kit (Beckman Coulter, California, USA) according to the manufacturer’s instructions. Sequences were corrected with CLC Genomics Workbench software (http://www.clcbio.com/) and drug resistance mutations were identified following the ANRS algorithm. Drug resistance test results were given to the physician in order to adjust the treatment regimens when needed.

### Phylogenetic analysis

Prot-RT concatemers nucleotide sequences were aligned with a set of different reference sequences representing HIV-1 sub-types obtained from the LANL database (http://www.hiv.lanl.gov) using CLUSTAL W. Phylogenetic trees were constructed using MEGA software: genetic distances were calculated with the Kimura two-parameter method, and trees were obtained by the neighbor-joining method. The reliability of the tree topology was estimated by bootstrap method with 1000 replicates. The Prot-RT concatemers nucleotide sequences were submitted to the GenBank database with accession numbers MW245238-MW245294.

### Main outcome and definition

Virological failure (VF) was the main outcome and defined as either two consecutive plasma viral loads >1000 copies/ml (3.0 log_10_ copies/ml) taken at least 6 months after ARV treatment initiation, or a VL>1000 copies/ml (3.0 log_10_ copies/ml) at the last available measurement, or death after at least 6 months of treatment [21]. Fifty-five children were excluded: children who didn’t start cART (n = 18) and those with cART duration less than 6 months due to deaths or lost to follow-up (n = 37). Therefore, a total of 155 children were considered for analyses.

When VL was >1000 copies/ml (3.0 log10 copies/ml), adherence support was provided to the caregiver, and VL control was carried out 2–3 months later. HIV drug resistance test was further performed when the control VL remained >1000 copies/ml (3.0 log10 copies/ml).

### Statistical analysis

Median and interquartile range were used for descriptive analysis of quantitative variables and percentages for categorical variables. The proportion of cases of virological failure was estimated with 95% confidence intervals. Chi squared and Fisher exact tests for categorical variables, and Student’s or Wilcoxon tests for continuous variables were used as appropriate to compare baseline characteristics between LPV/r-based and NVP-based regimen groups. A p-value of 5% was chosen as the threshold for statistical significance. Analyses were performed using R version 3.2.2 software.

## Results

### Characteristics of the study population at inclusion

A total of 210 HIV-infected children were included in the ANRS Pediacam cohort. Among them 14 deaths were recorded (1 before cART and 13 after cART initiation), 4 refused cART, and 37 received cART for a period less than six months, leaving 155 children considered in the final analysis. Description of baseline characteristics was performed only for the 155 remaining children that were considered for subsequent analyses (Table 1) as there was no significant difference with those excluded (n = 55). These baseline characteristics included: clinical sites, gender, socioeconomic level, immune status and VL at inclusion.

**Table 1:** Baseline characteristics of HIV-infected children and family at enrolment, ANRS-Pediacam cohort, 2007–2016.

Characteristics	N = 155
	n (%) or median (IQR)
Clinical sites	
MCC-CBF	74 (47.7)
LH	36 (23.2)
EHC	45 (29.0)
Gender	
Female	84 (54.2)
Mother’s age	29 (25–32)
Mother’s marital status (n = 152)	
Engaged	93 (60.0)
Single	59 (38.1)
Mother’s occupation (n = 152)	
Unemployed	77 (49.7)
Students	17 (11.0)
Worker	58 (37.4)
Mother’s level of education (N = 152)	
Out-of-school	51 (32.9)
Secondary school	91 (58.7)
Higher education	10 (6.5)
Refrigerator at home	68 (43.9)
Running water at home	76 (49.0)
Electricity at home	134 (86.5)
Weight-for-age Z-score (< -2)	57 (36.8)
Age at ART initiation	
< 4 months	70 (45.2)
≥ 4 months	85 (54.8)
Median (IQR)	4.2 (3.2–5.8)
ART regimen at inclusion	
AZT/3TC/LVP/r	76 (49.0)
3TC/D4T/LVP/r	27 (17.4)
AZT/3TC/NVP	40 (25.8)
3TC/D4T/NVP	11 (7.1)
Other: AZT/3TC/ABC	1 (0.6)
Immune status and VL at inclusion	
WHO stage I/II	99 (63.9)
WHO stage III/IV	56 (36.1)
Median CD4% (IQR)	23 (16–32)
Median VL (IQR)	6.5 (5.9–6.9)
Mother PMTCT exposure (N = 155)	
Not exposed	82 (50.3)
Exposed	73 (47.1)
• Mono or bitherapy	19 (26.0)*
• Tritherapy	54 (74.0)*
Unknown	4 (2.6)
Duration of mother PMTCT	
Median (months) (IQR)	2.2 (1.0–2.8)
Child PMTCT exposure at birth (N = 95)	
NVP alone	4 (4.2)
AZT alone	9 (9.5)
NVP+AZT	82 (86.3)
Anaemia at inclusion	18 (11.6)
Underweight at inclusion	57 (36.8)

MCC-CBF, Mother and Child Center of the Chantal Biya Foundation; EHC, Essos Hospital Center; LH, Laquintinie Hospital.

ART, antiretroviral therapy; PMTCT, prevention of mother-to-child transmission; VL, viral load; IQR, Interquartile range; 3TC, lamivudine/emtricitadine; AZT, zidovudine; D4T, stavudine; ABC, abacavir; NVP, nevirapine; LPV/r, Lopinavir/ritonavir.

*The percentages were calculated using N = 73 which is the total number of mothers exposed to PMTCT.

Of the 155 HIV-infected children considered, 66 children and their mothers received MTCT prophylaxis, 29 received prophylaxis but their mother did not, 7 did not receive prophylaxis while their mother did, in 49 cases neither the children nor their mothers received prophylaxis, and for 4 children no information on MTCT prophylaxis was available. Therefore, among the 155 HIV-infected children, 54.2% (84/155) children were female, 61.3% (95/155) received MTCT prophylaxis at birth and 73 (47.1%) were born from mothers who received MTCT prophylaxis consisting of different protocols as presented in Table 1. The median age at cART initiation was 4.2 months (interquartile range (IQR): 3.2–5.8), with LPV/r-containing regimen for 103 (66.5%) children, NVP for 51 (32.9%) and combination of three nucleoside inhibitors for 1 (0.6%) child. No difference was found concerning age at cART initiation between children with LPV/r and those with NVP-containing regimens (3.9 months (IQR: 3.2–5.4) *vs* 4.2 months (IQR: 3.2–5.6); p>0.05). At baseline, the median viral load was 6.5 log_10_ (IQR: 5.9–6.9) and the median CD4 percentage was 23 (IQR: 16–32). Overall, 76 (49%) children were initially treated with AZT/3TC/LVP/r, 27 (17.4%) with 3TC/D4T/LVP/r, 40 (25.8%) with AZT/3TC/NVP, and 11 (7.1%) with 3TC/D4T/NVP. Subsequently, some of these regimens changed during the course of follow-up (S1 and S2 Figs) due to stock out or change of national guidelines.

### Virological failure and drug resistance

#### Virological failure

After five years follow-up, 63 (40.6%; CI: 32.9–48.8) of the 155 children experienced virological failure (VF) at least once. The median duration between cART initiation and first VF was 22.1 months (IQR: 11.9–37.1) with a median VL of 4.8 log_10_ (IQR: 4.0–5.5) and median CD4% of 32 (IQR: 22–41). This duration was not different between children on NVP and LPV/r-containing regimens (20.6 (IQR: 10.4–34.9) *vs* 24.5 (IQR: 12.3–37.5); p-value = 0.35). The median duration between the first and second VL >1000 copies/ml was 4.14 months (IQR: 2.99–6.02). A total of thirty-three (52.4%) children experiencing VF had received MTCT prophylaxis at birth.

#### Resistance mutations

**Fig 1:**
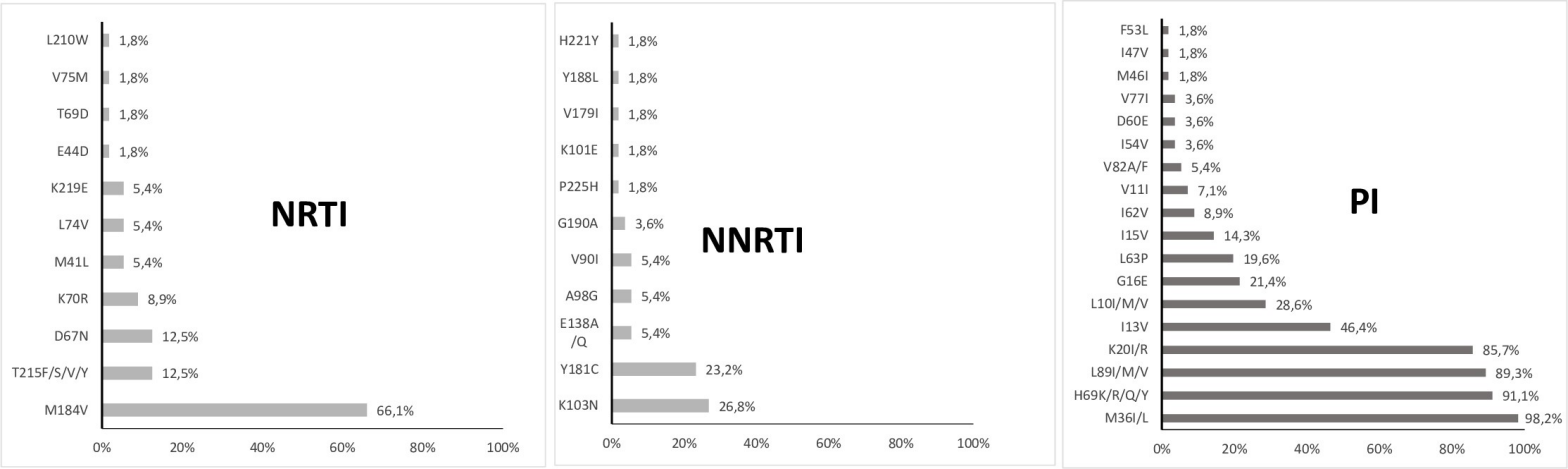
Drug resistance mutations detected in HIV-1 strains among children with virological failure. Drug resistance mutations are presented by increasing order of expression level. Mutations for different drug classes are indicated: NRTI (nucleoside reverse transcriptase inhibitors); NNRTI (non-nucleoside reverse transcriptase inhibitors); PI (protease inhibitors). V11I, K20I/R, M36I/L, H69K/R/Q/Y and L89I/M/V are considered polymorphic mutations present in vast majority of HIV-1 non-B subtypes.

Genotypic resistance testing was available for 57/63 (90.5%) children; samples were not available for two children and four samples failed to amplify due to low viral load, at the lower limit of the sensitivity of the assay (i.e., 3.3–3.6 log10 copies/ml). Among the 57 children with HIV drug resistance results, 40 (70.2%) had at least one drug resistance mutation (DRM). The predominant DRM among the NRTI was M184I/V (66.1%) followed by T215F/S/V/Y and D67N (12.5% each) and K70R (8.9%) (Fig 1). Conversely, fewer mutations were observed for: E44D, T69D, V75M and L210W at 1.8% each. Concerning the NNRTI, the predominant mutations were K103N (26.8%) and Y181C (23.2%) followed by V90I, A98G and E138A/Q (5.4% each). Regarding the PI, few (≤ 5%) major resistance mutations were found (V82A/F, I54V, M46I, I47V and F53L). However, polymorphism mutations naturally present in HIV-1 non-B subtypes were found in the vast majority of patients including M36I/L (98.2%), H69K/R/Q/Y (91.1%), L89I/M/V (89.3%) and K20I/R (85.7%) (Fig 1).

#### ART drug resistance

Overall, among the 57 children with resistance test result, 40 (70.2%) had resistance to at least one currently used ARV drug excluding Tipranavir. Of these 40 children, resistance to only one drug was observed in 9 children (22.5%), resistance to 2, 3 and more than three drugs was observed in 4 (10%), 9 (22.5) and 18 (45%) children respectively.

**Fig 2:**
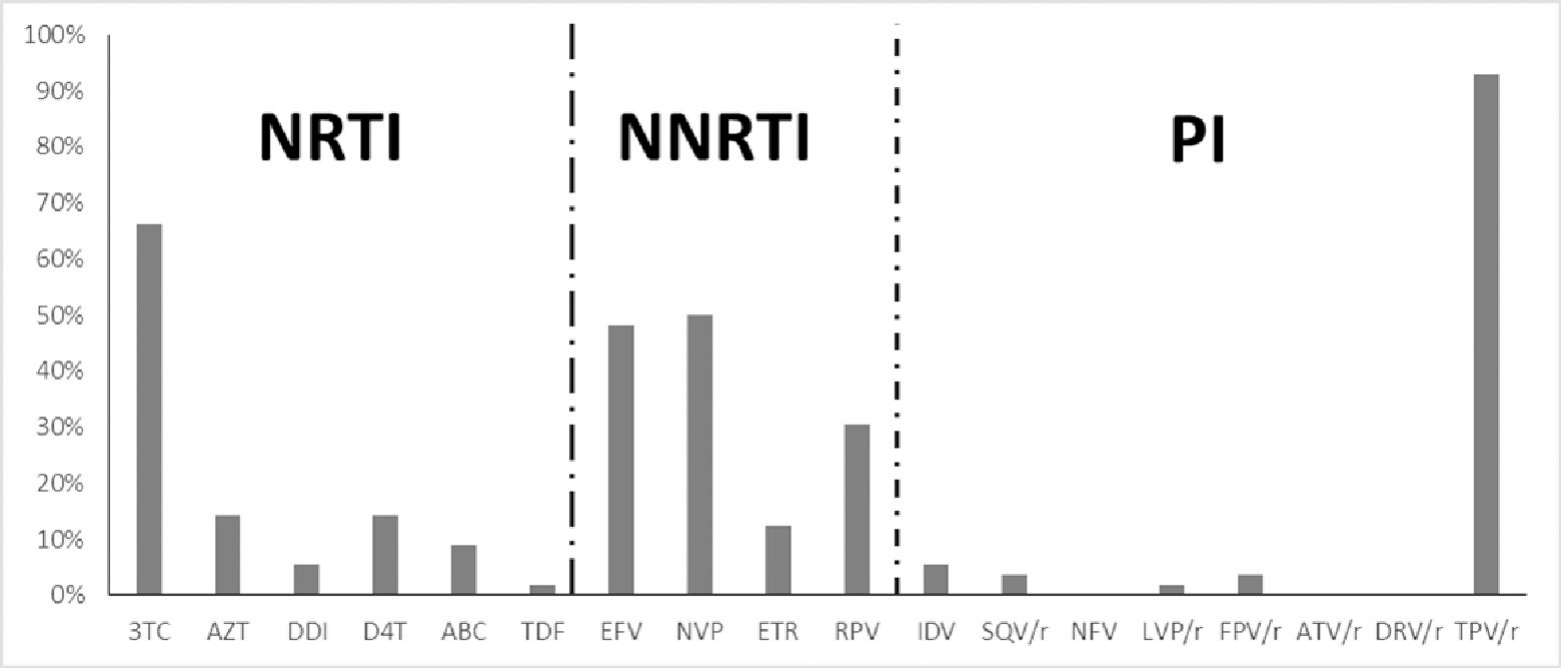
Resistance to different ARV drugs in children with virological failure. The proportion of resistance to individual ARV drug is shown on the figure. Different ARV classes are indicated: NRTI, nucleoside reverse transcriptase inhibitors; NNRTI, non-nucleoside reverse transcriptase inhibitors; PI, protease inhibitors. ARV drugs are shown on the X-axis and the percentage of detection on the Y-axis. 3TC/FTC, lamivudine/emtricitadine; ZDV/AZT, zidovudine; DDI, didanosine; D4T, stavudine; ABC, abacavir; TDF, tenofovir; EFV, efavirenz; NVP, nevirapine; ETR, etravirine; RPV, rilpivirine; IDV, indinavir; SQV, saquinavir; NFV, nelfinavir; LPV, lopinavir; FPV, fosamprenavir; ATV, atazanavir; DRV, darunavir; TPV, tipranavir; r, ritonavir.

Concerning the NRTI, about two third of patients showed resistance to 3TC (64.3%), resistance to AZT and d4T was observed in 14.3% patients and only one child (1.8%) was resistant to TDF (Fig 2). Among the NNRTI, the higher resistance rate was observed with NVP (50%) followed by EFV (48.2%). High proportions of resistance were obtained with the second generations NNRTIs namely RPV and ETR (30.4% and 12.5% respectively). Regarding the PI, almost all the children were susceptible except four children among which: three were resistant to IDV, two to SQV/r and FPV/r and one to LPV/r (Fig 2). Since HIV-1 non-B subtypes are naturally resistant to TPV, this drug was not included in the list.

We further assessed resistance according to the history of ART in children and we found that, all children with resistance to 3TC were either treated with the drug (Table 2); or were born from mothers treated with ART regimen containing 3TC (n = 13; 35.1%). About 75% of children with resistance to AZT were exposed to the drug before resistance developed. The same result was obtained with NVP where 78.6% of the children harbouring resistance to this drug were previously exposed to it. Similarly, the sole child with resistance to LPV/r was treated with this drug (Table 2). Conversely, only few or none of the children with resistance to EFV and ETR or RPV were exposed to the drug before (7.4 and 0% respectively).

**Table 2:** History of ART in children with drug resistance.

					ARV drugs						
	3TC	AZT	D4T	ABC	DDI	TDF	NVP	EFV	RPV	ETR	LPV/r
Resistance	37 (66.1%)	8 (14.3%)	8 (14.3%)	5 (8.9%)	3 (5.4%)	1 (1.8%)	28 (50%)	27 (48.2%)	17 (30.4%)	7 (12.5%)	1 (1.8%)
ART regimen containing the drug before resistance testing (child)^a^	37 (100%)	6 (75%)	2 (25%)	1 (20%)	0	0	22 (78.6%)	2 (7.4%)	0	0	1 (100%)
ART regimen containing the drug before resistance testing (mother)^b^	13 (35.1%)	1 (12.5%)	0	0	0	0	6 (21.4%)	1 (3.7%)	0	0	0

The percentage of resistance is given as the ratio of patient with the resistance to the drug relative to the total number of patients tested. The percentage of children (a) or mothers (b) exposed to a particular drug before resistance testing is given as the ratio of patient treated with the drug relative to the total number of patients experiencing resistance to the corresponding ARV drug. 3TC, lamivudine/emtricitadine; AZT, zidovudine; D4T, stavudine; ABC, abacavir; DDI, didanosine; TDF, tenofovir; NVP, nevirapine; EFV, efavirenz; RPV, rilpivirine; ETR, etravirine.

### Phylogenetic analyses

All the 57 children with drug resistance results were infected with HIV-1 non-B subtypes. The majority were classified as CRF_02 AG (68.4%) followed by subtypes G (10.5%), A (5.3%), and D (3.5%). The least represented subtypes were F2, CRF01_AE, CRF06_cpx, CRF11_cpx, CRF13_cpx and CRF022_01A1, all equally represented at 1.8% each.

## Discussion

This analysis was conducted within the framework of the ANRS-Pediacam cohort in order to assess the level of ART resistance among HIV-infected children initiating early cART at a median age of 4.2 months in three referral hospitals in Cameroon.

We found that after five years follow-up, about 41% of children experienced virological failure (VF) at least once. The high proportion of VF observed in our analysis has already been reported by Ateba *et al*. in the first two years of follow-up in the Pediacam cohort [16]. They found that the only factor associated with time to achieve virological success was good adherence (52% before two years follow-up) [16]. Other factors including poor adherence, age of patients, limited access to drug formulations adapted to paediatric needs, and treatment duration could be associated with VF. Also, younger age could be an additional factor related to VF in the Pediacam cohort when participants are aged less than 6 years as reported by other studies in Ethiopia and Kenya [22, 23].

As concerns drug resistance, overall, 70% of children with VF showed resistance to at least one ARV drug excluding TPV/r. In the NRTI class, the highest resistance rate was observed with 3TC due to the presence of M184I/V mutations as previously showed by other studies [18, 24–26]. It is well known that exposure to 3TC leads to a rapid selection of this mutation [26]. We observed that all the children harbouring resistance to 3TC were exposed to this drug through cART or their mother’s regimen. This could be also pointed out as further evidence of non-adherence in this study. However, M184V mutation could have clinical benefits since it is associated with impaired viral fitness, increased RT fidelity and hypersensitization to several other NRTIs principally AZT and TDF [26–28]. Resistance to other NRTIs (AZT, ABC and TDF) was rarely observed in our study, consistent with recent studies in Central African Republic and Malawi where viruses in more than 80% of children experiencing virological failure remained susceptible to AZT, ABC and TDF [24, 29]. These results support the WHO strategies to adopt NRTIs (especially AZT and ABC) in preferred and alternative cART regimens in children and neonates [30].

Regarding the NNRTI, the predominance of K103N and Y181C mutations was observed, which resulted in resistance to the routinely used NNRTI (NVP and EFV). This is consistent with other studies in Africa and is probably due to the extensive use of NVP for PMTCT [29, 31]. We found that almost all the children with resistance to NNRTI had history of exposure to these ARV drugs including their mother’s regimens. Similar findings were reported in South Africa (SAPMTCTE study), Swaziland and Zimbabwe [32, 33]. Since NVP is the core drug in PMTCT regimen, high level of resistance to this drug could therefore be due to persistent resistance in HIV-infected children who had failed MTCT prophylaxis [34]. This might support the recent WHO guidelines which adopted more effective protease and integrase inhibitors in first and second lines cART protocol in children [30].

We found low frequencies of resistance to PI namely IDV, SQV and FPV. Only one child was resistant to LPV/r which is the preferred PI for 1^st^ and 2^nd^ lines ART-regimens for children in RLS including Cameroon [6, 21]. This is consistent with results from the MONOD study where resistance to LPV/r was less than 5% (1/28) in children on LPV/r-based ART for 12 months [25]. Almost all the PI remained active in children of the ANRS Pediacam cohort after 5 years of treatment. However, more attention should be paid on treatment adherence to avoid accumulation of drug resistance mutations that could compromise long-term treatment in these children.

The high level of wild-type virus found in 30% of VF could be due to wrong declaration by caregivers concerning adherence resulting in unnecessary resistance testing. Although, genotypic resistance test is an interesting tool for HIV-infection treatment management, it will be of utmost importance to regularly clarify the process with health personnel for better optimization of its use.

Our study limitation is the lack of well-documented mother’s ART regimen files. Therefore, it was difficult to distinguish primary from secondary drug resistances. However, the major strength of our study is the optimal follow-up of early treated HIV-infected children for a long period. Our results highlight the challenges and concerns about routine follow-up of HIV–infected children and adults in RLS.

In Conclusion, we found a high proportion of virological failure (VF) in children on early cART follow-up with regular viral load (VL) testing for five years in the ANRS-Pediacam study. Majority of them harboured drug resistance mutations. These results emphasize the need to increase access to VL test in RLS for optimal follow-up of People Living With HIV under cART. Also, it will be very interesting to discuss how important is the role of the caregiver in achieving virological success or in assessing antiretroviral resistance.

## Supporting information

S1 Fig Flow-chart of therapeutic changed in children with initial cART containing LPV/r. (TIF)

S2 Fig Flow-chart of therapeutic changed in children with initial cART containing NVP. (TIF)
