# Supplementary figures and images for: Virological failure and antiretroviral resistance among HIV-infected children after five years follow-up in the ANRS 12225-PEDIACAM cohort in Cameroon
Source: PLoS One. 2021 Mar 18;16(3):e0248642. doi: 10.1371/journal.pone.0248642 (PMC7971859; doi:10.1371/journal.pone.0248642)

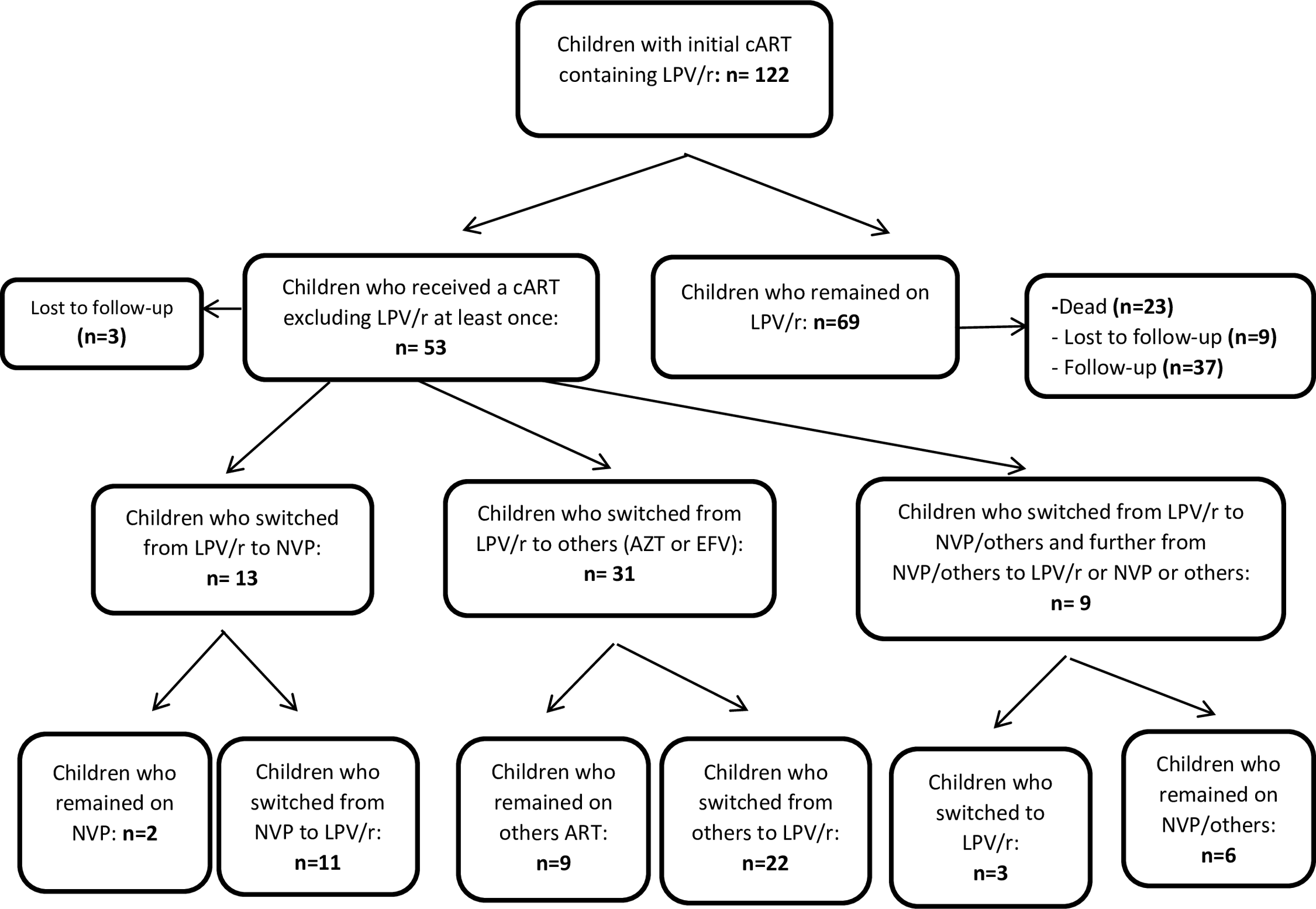

Supplement: S1 Fig — (TIF) [file pone.0248642.s001.tif]

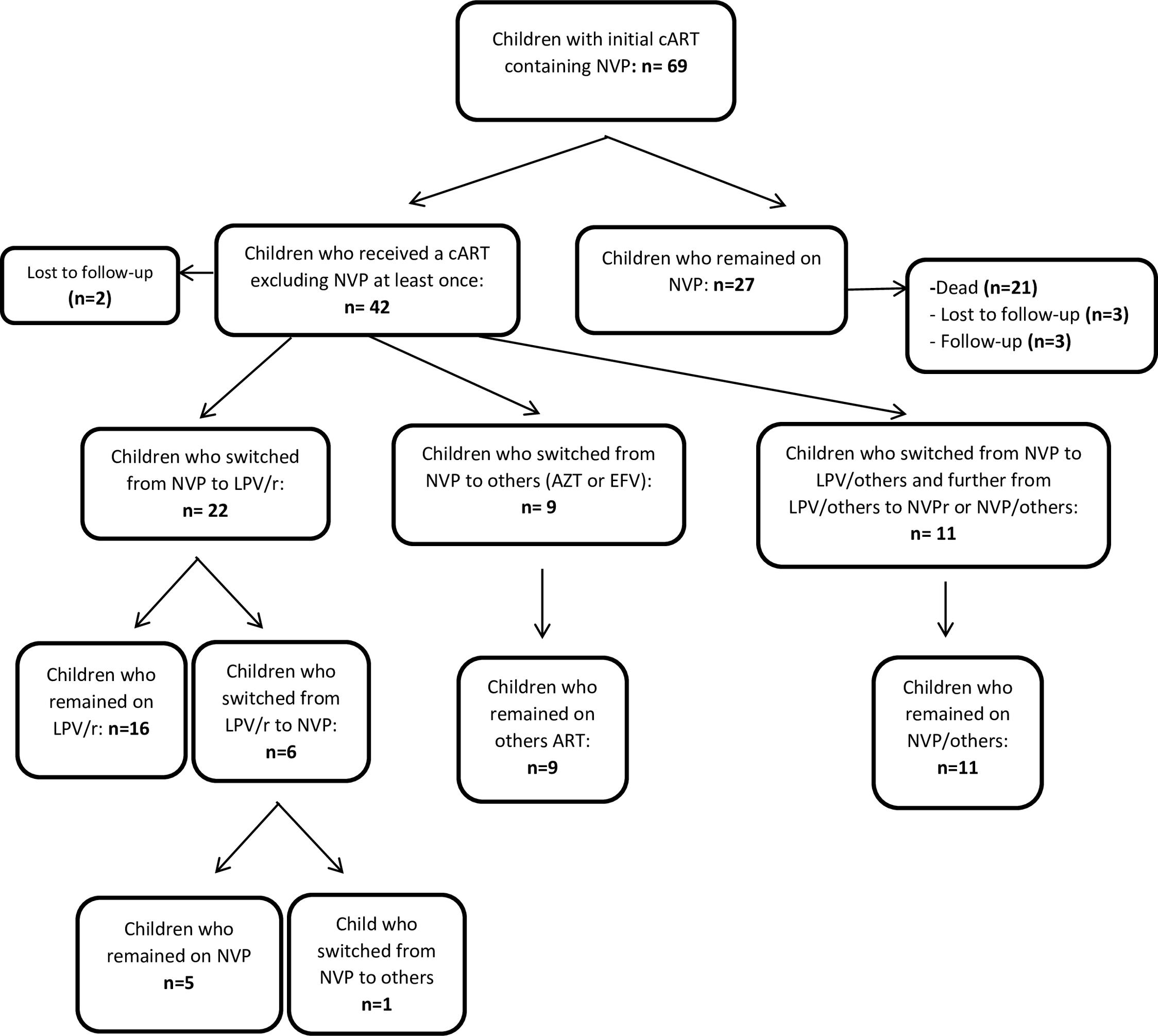

Supplement: S2 Fig — (TIF) [file pone.0248642.s002.tif]
